# Salvage minimally invasive robotic and laparoscopic pyeloplasty in adults: a systematic review

**DOI:** 10.1080/2090598X.2022.2082208

**Published:** 2022-06-28

**Authors:** Mai Elaarag, Hind Alashi, Maya Aldeeb, Ibrahim Khalil, Ahmad R. Al-Qudimat, Abdelhamed Mansour, Abdulla A Al-Ansari, Omar M. Aboumarzouk

**Affiliations:** a Surgical Research Section, Department of Surgery, Hamad Medical Corporation, Doha, Qatar; b Hamad General Hospital, Hamad Medical Corporation, Qatar, Doha; c College of Medicine, Qatar University, Doha, Qatar; d Dentistry and Nursing, the University of Glasgow, Glasgow, UK

**Keywords:** Salvage, secondary, robotic, laparoscopic, pyeloplasty, adults

## Abstract

**Introduction:**

A UPJO is a blockage of the ureter that affects urine flow. UPJO is mainly treated by an open approach, however, in recent years minimally invasive techniques are taking place. These techniques include robotic and laparoscopic pyeloplasty. Some patients require a redo after a primary intervention. A systematic review was conducted through the examinations of the efficacy and safety of a robotic redo pyeloplasty in adult patients from previous literature reviews.

**Methods:**

A literature search was made through PubMed. A selection process was done based on our eligibility criteria. The data were represented numerically, listed on tables and analyzed cumulatively using Microsoft Excel.

**Results:**

Twenty studies were included in this review, of which nine were studies on robotic outcomes () (157 patients), 10 on laparoscopic (210 patients), and one review by Zhang et al., focused on both types of surgeries. Two papers (24 patients) from the robotic studies and one paper (21 patients) from the laparoscopic studies were excluded from the intra and post-operative characteristics because not enough data were available and were only included for the success and complication rates. The success rate for the robotic studies was 88.5% while the laparoscopic studies had a success rate of 91%. However, the robotic studies had a complication rate of (11.8%) while the laparoscopic studies had a complication rate of (15.9%). Conversion surgery was required in one patient undergoing laparoscopic surgery.

**Conclusion:**

The minimally invasive methods are becoming more viable in adult patients with rUPJO, considering its effectiveness and fast recovery. This can lead to a new era of robotic assisted surgeries to becoming the gold standard.

**Abbreviations:** Systematic review: Redo robotic and laparoscopic pyeloplasty in adults; UPJO = Ureteropelvic junction obstruction; rUPJO = redo ureteropelvic junction obstruction.

## Introduction

Ureteropelvic junction obstruction (UPJO) is a condition caused by an obstructed segment of the ureter, leading to failure of antegrade flow of urine[1]. Most cases are congenital, however, can occur due to stenosis of the UPJO following endoscopic procedures, such as laser lithotripsy or tumor ablation. Though many cases are diagnosed in early years of life and undergo corrective surgery, some cases are diagnosed in adulthood. The standard treatment modality is a pyeloplasty and has a high success rate of 90–100% [2–6].

Traditionally, pyeloplasty repairs have been carried out with the open technique, however, laparoscopic repair has overtaken the open technique with similar postoperative outcome results. First described in 1993, since then, laparoscopic pyeloplasty has almost exclusively replaced the open technique in centers where an experienced laparoscopic surgeon is present [6–9]. Laparoscopy gives the advantage of minimally invasive surgery with reduced hospital stay, less pain, and blood loss, and maintains the high success rate of the open procedure. Laparoscopy is designed with a 2D vision and the rotation is limited, this is because the arms are directly used by the surgeon and involves visuomotor rotations [10].

**Figure 1.**
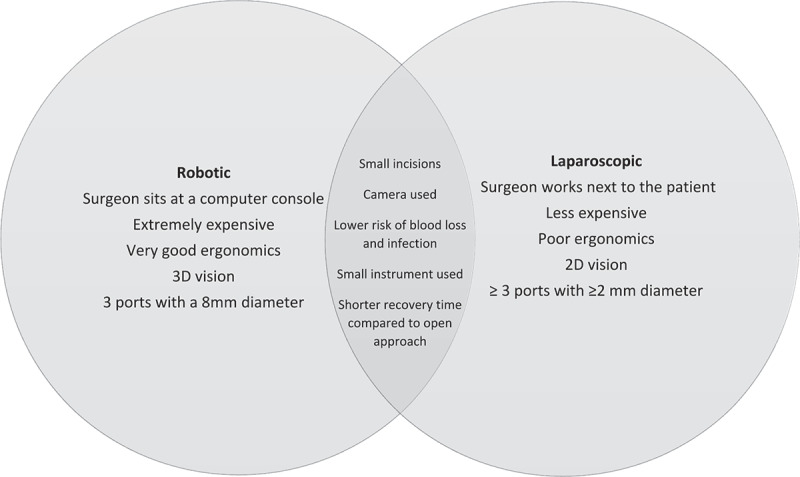
Venn diagram comparing the main differences and similarities between robotic and laparoscopic surgeries.

Robotic assisted pyeloplasty was first approved in 2000 and has helped to overcome some limitations of the conventional laparoscopic approach [11]. For instance, it was designed with a magnified 3D vision and provides articulating robotic arms that offer 7 degrees of freedom and has a built-in tremor-filtration technology [12]. Since then, the robotic technique has slowly but progressively replaced most laparoscopic procedures in urological surgery. Studies have emerged reporting their experiences of various robotic operations. Generally, the main differences between robotic and laparoscopic surgeries are the expenses, level of ergonomics, number/diameter of ports, and the surgeons’ site while performing the operation [13]. Figure 1 represents the major differences and similarities between the two procedures [14].

Despite multiple reviews published on the topic, there is a lack of clarity to the success rate and complication rates of salvage or redo pyeloplasty in the adult population with the minimally invasive techniques. As the pediatric population carries a different approach, given the technical difficulties in operating on small children compared to full-grown adults in addition to the long learning curve, the success rate of these procedures conducted in adult life might carry a different operative outcome [15,16]. Therefore, we aimed to conduct a systematic review of the literature to determine the safety and efficacy of salvage or redo laparoscopic and robotic pyeloplasty after a failed first attempt of repair in adults.

## Methods

### Search strategy

The systematic search of the literature was performed using Cochrane and Prisma Guidelines [17,18]. To identify relevant articles, the search strategy included the following databases: The US National Library of Medicine’s life science database (MEDLINE) (1990-May 2021), EMBASE (1990-May 2021), Cochrane Central Register of Controlled Trials – CENTRAL (in the Cochrane Library – 2021), CONAHL (1990-May 2021), Clinicaltrials.gov, Google Scholar, and Individual urological journals.

The search terms used: Laparoscopy, Laparoscopic, Robotic,Pyeloplasty, Ureteropelvic Junction Obstruction, Salvage, Secondary, Redo, and Adults.

Phrases used for the Medical Subject Heading [MeSH] search included: (((“Pyeloplasty’ [MeSH]) AND ‘Adult’ [MeSH]) AND ‘Redo’ [MeSH]) OR ‘Secondary’ [MeSH] OR ‘Salvage’ [MeSH], ‘Pyeloplasty’(Mesh) AND ‘Laparoscopy’(Mesh), and ‘Robotic’(Mesh) AND ‘Kidney Pelvis’(Mesh).

### Study selection

All published articles that looked at the outcomes of salvage, secondary, or tertiary laparoscopic pyeloplasty in adults were included. Authors were contacted whenever the data were not available or clear. If data were not extractable, provided, or clarified, the study was excluded. If data was mixed with pediatric cohort and cannot be distinguished from the adult cohort, the study was excluded.

Three reviewers (ME, HA, OA), independently identified studies that appeared to fit the inclusion. Disagreement between the authors in the study was resolved by consensus.

Only published studies reporting outcomes of salvage laparoscopic and robotic pyeloplasty in adult population were included.

Salvage was defined as a definitive procedure after a failed attempted repair of the UPJO with either previous open, laparoscopic, or endoscopic operations.

### Eligibility criteria

The papers included in this review were mainly focused on adult patients who underwent a redo robotic pyeloplasty and laparoscopic pyeloplasty regardless of the primary intervention. The aim was to extract and analyze the efficacy and safety of this surgery in the two surgical approaches.

### Data extraction and analysis

Two authors (ME and HA) independently extracted the data of each included study, the senior author (OA) reviewed the data extracted to ensure quality assurance of data. Discrepancy of the data extraction was resolved by consensus.

The main variable that we were aiming to extract was the success rate. However, other pre-operative and postoperative variables were extracted. These variables include number of patients, age, operative time, blood loss, length of hospital stay, follow-up duration, total complication, and conversion rates.

We conducted a cumulative analysis to determine the overall percentage of results. Where appropriate, either mean and standard deviation or summation and expressed as percentage (%).

T-test was used to find the correlation of the two surgeries and their complication and success rate using SPSS V21 program.

## Results

### Characteristics of included studies

For the robotic procedures, a flow diagram is shown in Figure 1. Primarily, 110 papers were included from the literature search. Seventy papers were excluded after the screening and 27 were potentially illegible for a full manuscript evaluation. In total, 10 papers met our inclusion criteria [11,12,19–26]. Table 1 illustrates the basic characteristics of each study.

**Table 1.** Intra and post-operative characteristics of robotic studies.

Author, yr.	No. of patients	Mean age (years), (range)	Mean operative time (mins), (range)	Mean blood loss, (ml), (range)	Mean hospital stay (days), (range)	Mean follow up (months), (range)
Lee, 2020^[20]^	28	44*(31–57)	188* (135–226.5)	100* (50–175)	1* (1–2)	20.3* (9.3–25.3)
Dirie, 2021^[11]^	13	25* (18–51)	100* (0–300)	148* (79–308)	6 (3–14)	25 (15–56)
Thom, 2012^[25]^	9	NM	205 (144–433)	125 (25–800)	NM	NM
Atug, 2006^[39]^	7	37.8 (17–67)	279.8 (230–414)	52.5 (20–100)	1.2 (1–3)	10.7 (3–20)
Mufarrij, 2008^[22]^	23	40 (18–72)	215.96 (110–345)	68.3 (10–300)	2.1 (1–3)	24.1 (5–51)
Sivaraman, 2012^[12]^	21	36 (19–71)	190.4 (110–342)	86.2 (20–200)	1.7 (1–7)	NM
Niver, 2012^[23]^	17	41.8 ± 18.3	217.9 ± 52.5	98.8 ± 74.5	2.8	26 (17.43)
Zhang, 2019^[34]^	15	30.33 ± 13.26	126 ± 39	< 100	7.8 ± 3.1	16.93 ± 8.63
Total/Averages	**133.00**	**37.19**	**205.84**	**86.16**	**3.60**	**20.55**

Median*, NM: Not mentioned, ± SD.

**Figure 2.**
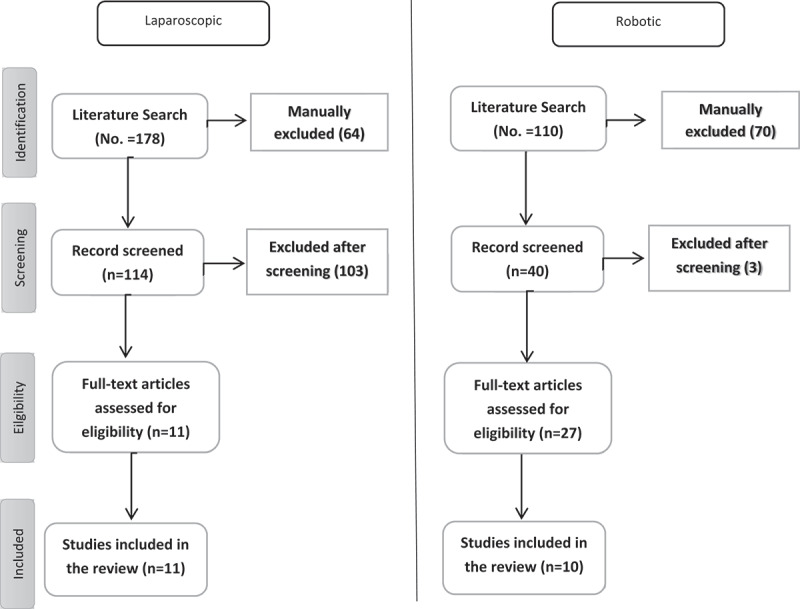
PRISMA diagram for article selection process.

For laparoscopic procedures, the literature search identified 178 studies (Figure 2). Of which 64 were excluded based on their titles and 103 papers were excluded after reviewing the abstracts, either because they exclusively reported the pediatric population or had mixed pediatric and adult results. Full manuscripts were evaluated in 11 studies. All 11 were included in our paper [5,7,26–34]. The studies were all published within the last 20 years reflecting current evidence. There was a total of 367 patients who underwent a redo or salvage robotic or laparoscopic pyeloplasty for UPJO obstruction.

Most of the studies used were retrospectively conducted, while a few were both retrospectively and prospectively analyzed on adult patients who underwent redo pyeloplasty for rUPJO.

Some characteristics that we were specifically looking for were not mentioned in most of the studies, therefore they were excluded from the data analysis.

### Cumulative analysis

In the robotic studies, there is a total of 157 adult patients included in the success and complication rates presented in Table 3. Two studies (24 patients) were excluded from the intra and post-operative characteristics because not enough data were available, which brings the total number of patients in Table 2 to be 133. The average age was 37.19 ranging between 17 and 72.

**Table 2.** Intra and post-operative characteristics of laparoscopic studies.

Author, yr.	No. of patients	Mean age (years), (range)	Mean operative Time (mins), (range)	Mean blood loss (ml), (range)	Mean hospital stay (days), (range)	Mean follow up (months), (range)
Hammady, 2017^[7]^	32	29 ± 6 (21–45)	133 ± 42 (100–185)	55 ± 36 (30–190)	2.7 ± 2.3 (2–6)	32.4 ± 14 (19–48)
Eden, 2004^[30]^	11	NM	173.3	NM	2.8	20.2
Swearingen, 2016^[5]^ (secondary procedure)	3	51 ± 8	199 (123–315)	83 (25–175)	1.3 (1–2)	20* (15–21)
Swearingen, 2016^[5]^(tertiary procedure)	12	29 ± 14	209 (135–270)	145 (25–600)	3 (1–7)	20* (1–75)
Brito, 2007^[28]^	13	34.9 (18 − 44)	195 (135–270)	NM	2.2 (2–5)	22.4 (16–36)
Sundaram, 2003^[33]^	36	34 (16–60)	372 (162–600)	67 (25–200)	2.9 (1–7)	21.8 (3–85)
Shapiro, 2009^[26]^	9	30.5 (19–50)	204 (80–264)	105 (20–300)	2.1 (2–3)	62.6 (6–124)
Shadpour, 2011^[32]^	11	41.4 (27–55)	208 (165–250)	NM	3.6 (3–5)	24.1 (12–24)
Chiancone, 2017^[29]^	38	26.6 ± 6.5	103.16 ± 30	122.37 ± 73.25	4.47 ± 0.86	42.5 ± 24.6
Zhang, 2019^[34]^	14	34.71 ± 10.5	193.8 ± 30	<100	9.79 ± 1.76	30.43 ± 12.91
Ambani, 2017^[27]^	10	39.6 (41.0)	246.8 (159–415)	110 (25–175)	2.4	18.6*
Total/Averages	**189.00**	**35.07**	**203.37**	**98.20**	**3.39**	**30.71**

Median*, NM: Not mentioned, ± SD.

**Table 3.** Success, complication rates and number of conversions for robotics and laparoscopic studies.

Author, yr.	Type of surgery	No. of patients	Success rate, n (%)	Complication rate, n (%)
Lucas, 2012^[21]^	Robotic	12	10 (83.3%)	2 (16.7%)
Lee, 2020^[20]^	Robotic	28	24 (85.7%)	0
Dirie, 2021^[11]^	Robotic	13	10 (76.9%)	3 (23.1%)
Thom, 2012^[25]^	Robotic	9	7 (77.8%)	NM
Atug, 2006^[39]^	Robotic	7	7 (100%)	0
Schwentner, 2007^[24]^	Robotic	12	11 (91.7%)	NM
Mufarrij, 2008^[22]^	Robotic	23	21 (91.3%)	2 (9.52%)
Sivaraman, 2012^[12]^	Robotic	21	20 (95.2%)	3 (14.3%)
Niver, 2012^[23]^	Robotic	17	16 (94.1%)	3 (17.6%)
Zhang, 2019^[34]^	Robotic	15	13 (86.7%)	3 (20%)
Hammady, 2017^[7]^	Laparoscopic	32	29 (90.6%)	7 (21.9%)
Eden, 2004^[30]^	Laparoscopic	11	10 (90.9%)	1 (9.1%)
Swearingen, 2016^[5]^	Laparoscopic	15	15 (100%)	3 (25%)
Brito, 2007^[28]^	Laparoscopic	13	12 (92.3%)	0
Sundaram, 2003[^33]^	Laparoscopic	36	30 (83.3%)	8 (22%)
Ost, 2005^[31]^	Laparoscopic	21	20 (95.2%)	NM
Shapiro, 2009^[26]^	Laparoscopic	9	8 (88.8%)	1 (11.1%)
Shadpour, 2011^[32]^	Laparoscopic	11	10 (90.9%)	1 (9.09%)
Chiancone, 2017[^29]^	Laparoscopic	38	35 (92.1%)	6 (15.7%)
Zhang, 2019^[34]^	Laparoscopic	14	12 (85.7%)	2 (14.2%)
Ambani, 2017^[27]^	Laparoscopic	10	10 (100%)	1 (10%)

NM: Not mentioned

For the laparoscopic studies, there is a total of 210 patients included in the success and complication rates presented in Table 3. One study (21 patients) was excluded from the intra and post-operative characteristics because not enough data were available, which brings the total number of patients in Table 2 to be 189. The average age was 35.07. The mean operative time was 205.84 minutes. Average blood loss was 86.16 mL (10–300 mL). Hospital stays ranged between 1 and 14 days and on average, patients’ hospital stay was 3.6 days. The mean follow-up period for patients was 20.55 months ranging from 3 to 56 months.

As illustrated in Table 2, for laparoscopic surgery review, there was a total of 189 patients with a mean age of 35.07 (16 − 60) were analyzed. The mean operative time was 203.4 minutes (80–600), the average blood loss was 98.2 mL (20–300 mL), the hospital stay was 3.4 days (1–7 days), the average follow-up period was 30.7 months ranging from 1 to 124 months. [5,7,26–34]

### Efficacy of surgery

The total success rate was 88.5% for the robotic studies and 91% for the laparoscopic studies. Conversion surgery was required in only one patient undergoing laparoscopic surgery.

### Safety of surgery

The total complication rate in the robotic studies was 11.8%, while 15.9% in the laparoscopic studies.

## Discussion

UPJO is a medical condition that is characterized by stopping or slowing down the urine flow. Patients who suffer from UPJO are likely to develop progressive deterioration of renal function induced by increasing pressure and hydronephrosis of the kidney. A surgical intervention is recommended based on the differential renal function along with the pertinent anatomy and the degree of obstruction [35].

In 1891, the first successful surgical management for UPJO was announced. Consequently, different techniques were developed upon that. UPJO is treated with an open approach despite the paradigm shift that emerged through the establishment of minimally invasive methods. Minimally invasive methods include laparoscopic pyeloplasty which was introduced in 1993 and robotic-assisted laparoscopic pyeloplasty introduced in 2000. Both approaches have shown to be highly successful and effective [11,36,37].

Despite the high success rate, up to 10% of cases fail initial management and require a secondary or salvaged procedure to alleviate the obstruction or symptoms within the first year after primary repair [1,6]. Salvage procedure include endopyelotomy, robotic, laparoscopic or even open repair. As many of the cases are diagnosed early in life, many studies have reported their salvage repair success rates in the pediatric population [6,37].

The redo pyeloplasty can be challenging due to the previous surgery done. The challenges include, decreased vascularity to the obstruction area and scar tissue [11,37]. The success of the robotic or laparoscopic approach was not limited only by the patients’ fast recovery but also by the surgeons’ learning curve associated with it. In robotic surgeries, unexperienced surgeons tend to have short learning curves while still maintaining high-level performance when compared to laparoscopic surgery [12]. Nonetheless, due to the high success rates and low complication rates conducted by many individual studies, many hospitals are performing large numbers of robotic assisted pyeloplasty for UPJO patients as standard [6,21].

### Summary of main results

The main focus of our paper is the efficacy and safety of redo robotic and laparoscopic pyeloplasty in adult patients. Results from our study indicate that a redo or salvage pyeloplasty for the management of rUPJO is very effective. No statistically significant association were found between the success rate (p = 0.082, 95% CI −5.6–9.6) and failure rate (p = 0.292, 95% CI −5.4,1.11) in robotic and laparoscopic surgeries (Table 3). The studies included in our analysis had success rates ranging between 77% and 100% with an average success rate of 88.5% in the robotic studies and 93.2% in the laparoscopic studies.

Statistically significant associations (p = 0.028, 95% CI −1.6, 3.05, effect size = 0.72) were found in type of surgery (robotic and laparoscopic) with complication rate, 11.8% in the robotic studies and 15.9% in the laparoscopic studies. Although the high rate of success is slightly offset by the high complication rates, one must remember that these procedures are inherently more difficult to carry out because they redo surgeries. Therefore, a slightly higher complication rate than the initial procedure is somewhat expected. Nonetheless, the authors of all the studies do mention that all the patients recovered from their complications. However, as specific individual complication where not mentioned in most of the studies, we were unable to itemize these complications into table format or classify to Clavien-Dindo classification.

Generally, a redo pyeloplasty is considered more challenging because of the scar tissue, fibrosis, and adhesions that occur due to a previous operation [6] especially, when the primary procedure is an open surgery [38]. This has significantly impacted the operative time in redo operations as well as complications are more likely. Atug et al. from the robotic group and Sundaram et al. from the laparoscopic group supports this by reporting a higher operative time in the redo group [32,39]. However, robotic and laparoscopic pyeloplasty has been associated with shorter hospital stays when compared to open surgery [11,24,37]. This is crucial because longer hospital stays tend to increase the risk of hospital-acquired infections. In addition, as stated earlier, with increased skills and experience, operative time may potentially shorten as well. This all adds up to the success of robotic and laparoscopic surgeries compared to open surgeries.

### Advantages & implications for practice

Minimally invasive pyeloplasty has developed advantages, including a more quick and proficient learning curve for surgeons, furthermore, with the robotic technique the potential for unrivaled outcomes through the developed visualization and improved control [6]. Through proper structured training by increasing nursing, surgeon and anesthesiologist practice for minimally invasive pyeloplasty with a committed team can accomplish enduring results similar to the gold standard open surgery [40], leading to a shorter learning curve for the procedures [12].

Utilizing the robotic technique, there is less tremor motor movements as well as an unchallenging spatulation of the ureter while doing the anastomotic repair compared to laparoscopic [10]. In addition to more precise laying down of sutures to allow for optimal suture closure. Nonetheless, both techniques have shown to have a quicker recovery period, less blood loss, and shorter hospital stay than the classical open technique [6,10,11,37].

As shown by comparative studies, minimally invasive pyeloplasty is a successful alternative for managing either primary UPJO or rUPJO [6]. This leads to the minimally invasive approaches becoming the gold standard instead of the classic open approach. Furthermore, given the advantages of robotic surgery over conventional laparoscopic surgery, robotic pyeloplasty, and redo pyeloplasty has now become standard across many centers [6,10,11,21,37].

This is the first review to look at and analyze the literature specifically looking at robotic and laparoscopic redo pyeloplasty procedures for adult patients and can be used as a benchmark to help counsel patients during the consultation for the procedure.

### Disadvantages

Several disadvantages were stated in different studies. One of them is the high cost of robotic assisted interventions. Another one is the chance of damage or suture breakage that could result from the loss of tactile sensation along with the steep learning curve associated with intracorporeal suturing. Also, the interfering robotic arm in small-sized patients is an obstacle to some surgeons [41]. Nonetheless, these disadvantages can be overcome with experience and with high number of cases performed by centers, may offset the high cost. However, no study is available to determine numerical representation of cost comparing different techniques. Nonetheless, with the advent of different robotic machines, costing of the procedure will ultimately become more competitive and lessen.

### Limitation of our review

The main limitation was the relatively small number of reported redo/salvage cohort. However, these were representations of each studies’ practice, there was no reporting bias detected. Nonetheless, from a review point of view, these cases represent the existing literature that we can draw up cumulative results from. Furthermore, this review was conducted in a methodical protocol manner to ensure no reviewer bias was introduced.

### Implication for research

Further multicentered studies comparing between laparoscopic and robotic pyeloplasty can draw some crucial evidence for determining the best approach to treat patients with rUPJO. The 20 papers included in our analysis are the only articles that discuss redo pyeloplasty in the adult population of patients. The articles that were excluded from our analysis were excluded because they either focused on pediatric patients or primary pyeloplasty only. However, research is emerging in robotics and is becoming the preferred procedure in the treatment of different urological diseases. Therefore, as more centers adopt robotic or laparoscopic, more studies will emerge on the subject matter. Hence, coordinating and cooperation between centers to expand the evidence outcomes is recommended. Nonetheless, there remains more centers carrying out the robot-assisted procedures; hence, both minimally invasive techniques will continue to be an available option for patients.

## Conclusion

Our review has found that redo minimally invasive pyeloplasty is efficient with an 89–93% success rate utilizing either robotic or laparoscopic approaches, however, minimally invasive techniques for the treatment of UPJO dispenses promising results.
